# Effects of *In Vitro* Polyploidization on Agronomic Characteristics and Fruit Carotenoid Content; Implications for Banana Genetic Improvement

**DOI:** 10.3389/fpls.2019.01450

**Published:** 2019-11-12

**Authors:** Delphine Amah, Angeline van Biljon, Bussie Maziya-Dixon, Maryke Labuschagne, Rony Swennen

**Affiliations:** ^1^Plantain and Banana Improvement Program, International Institute of Tropical Agriculture, Ibadan, Nigeria; ^2^Department of Plant Sciences (Plant Breeding), University of the Free State, Bloemfontein, South Africa; ^3^Food and Nutrition Sciences Laboratory, International Institute of Tropical Agriculture, Ibadan, Nigeria; ^4^International Institute of Tropical Agriculture, The Nelson Mandela African Institution of Science and Technology, Arusha, Tanzania; ^5^Laboratory of Tropical Crop Improvement, Bioversity International, Heverlee, Belgium; ^6^Department of Biosystems, KU Leuven, Heverlee, Belgium

**Keywords:** banana genetic improvement, biofortification, carotenoid content, diploid bananas, *in vitro* polyploidization, vitamin A, pollen viability

## Abstract

Bananas (*Musa* spp.), native to South East Asia, have spread worldwide and are integrated into the diets of millions of people in tropical regions. Carotenoid content varies dramatically between different banana genotypes, providing an opportunity for vitamin A biofortification. Polyploidization is a useful tool for crop improvement with potential to generate new diversity, especially in a polyploid crop like bananas. Ten induced tetraploids generated from six diploid banana genotypes were evaluated for their agronomic attributes and fruit carotenoid content in comparison to their diploid progenitors. Tetraploids had distinct plant morphology, but generally displayed inferior vegetative and yield characteristics with 20% lower bunch weights than their original diploids. Similarly, a 50% decrease in fruit provitamin A carotenoids (α-carotene, 13-*cis* β-carotene, 9-*cis* β-carotene, *trans*-β-carotene) accompanied by a corresponding increase in lutein was recorded in induced tetraploids in comparison to their original diploids. Additionally, all lines were subjected to pollen viability tests to assess their fertility. Pollen viability tests indicated over 70% viability for induced tetraploids and diploid controls, suggesting their possible use in crosses. These findings provide a basis for the application of induced polyploidization in bananas to generate useful genetic material for integration in hybridization programmes aiming to produce vitamin A enriched triploids valuable to malnourished populations.

## Introduction

Micronutrient deficiency, also known as “hidden hunger,” is a major public health concern, caused by inadequate dietary intake of essential nutrients or minerals. One in three people worldwide are malnourished, with two billion people lacking key micronutrients, like iron and vitamin A (Global Nutrition Report, 2017). Traditional strategies, such as mineral supplementation and food fortification, have failed to completely eradicate micronutrient deficiencies due to lack of economic, social, and cultural mechanisms for efficient implementation. Recently, crop improvement efforts have focused on producing nutrient-rich high yielding crops through biofortification, as a sustainable solution to address micronutrient malnutrition (Bouis and Welch, 2010; Garg et al., 2018). Biofortification involves the use of conventional breeding or transgenic approaches to enhance the nutritional value of crops. Conventional breeding, which exploits natural genetic variability has been the preferred method for total carotenoids with vitamin A activity (pVAC) enhancement of sweet potato, maize, and cassava, with biofortified varieties already available in farmers’ fields (Bouis and Saltzman, 2017; Garg et al., 2018).

Bananas (*Musa* spp.), native to South East Asia, have spread worldwide and are integrated into the diets of millions of people in tropical regions. Most banana cultivars are derived from intra- and inter-specific hybridization of two wild diploid ancestral species, *Musa acuminata* Colla (A genome) and *Musa balbisiana* Colla (B genome), although *Musa schizocarpa* (S genome) and *Musa textilis* (T genome) are also reported to have contributed to the origin of some cultivars (Heslop-Harrison and Schwarzacher, 2007; Häkkinen, 2013; Christelová et al., 2017; Němečková et al., 2018). Based on ploidy and genome distribution, bananas are classified as diploid (AA and AB), triploid (AAA, AAB, and ABB), or tetraploid (AAAA, AAAB, and AABB) (Heslop-Harrison and Schwarzacher, 2007). Most important cultivars for food security are of the triploid category comprising dessert bananas (AAA and AAB), plantains (AAB), East African highland bananas (EAHB) (AAA-EA) and the Bluggoe and Pisang awak cooking types (ABB), hence the focus of banana breeding programs.

Besides being a principal source of calories, some bananas are a good source of micronutrients such as vitamin A, which can be enhanced by biofortification. Banana cultivars exhibit considerable genetic variability for pVACs, with some cultivars accumulating up to 35 µg g^−1^ FW of fruit pulp (Davey et al., 2009; Borges et al., 2014; Heng et al., 2017; Amah et al., 2019), but this variability has not been fully exploited in breeding. Banana breeding strategies rely on sexual hybridization across ploidy categories and phenotypic selection from viable offspring. The predominant breeding scheme involves crosses between 3*x* cultivars and 2*x* wild or improved accessions, selecting 4*x* and 2*x* hybrids from intermediate products and crossing selected 4*x* and 2*x* hybrids to generate sterile 3*x* hybrids (Tenkouano et al., 2011; Ortiz and Swennen, 2014; Brown et al., 2017). However, this process is relatively slow and is further complicated by parthenocarpy, polyploidy, and associated irregular meiotic behavior, low fertility, low seed viability, long generation times, diverse genome configurations, and the narrow genetic base (Ortiz, 2013; Brown et al., 2017; Batte et al., 2019). Nevertheless, triploid hybrids have been efficiently obtained using the 4*x*–2*x* scheme making tetraploids vital to 3*x* breeding.

Polyploidization has been advocated as a tool for genetic improvement in several crops with the aim of creating new polyploid varieties or increasing fertility of interspecific hybrids (Dhooghe et al., 2011; Sattlser et al., 2016; Denaeghel et al., 2018; Parsons et al., 2019). Polyploid induction has been proposed as an alternative breeding approach for bananas. This strategy aims to create triploids directly from doubled ancestral diploid cultivars through reconstitutive breeding (Bakry et al., 2009; Tenkouano et al., 2011). Polyploid induction in *Musa* spp. is achieved through treatment of highly proliferating totipotent explants with anti-mitotic compounds (colchicine or oryzalin), which interfere with microtubule formation, followed by plantlet regeneration and isolation of induced tetraploids through flow cytometry (Hamill et al., 1992; Van Duren et al., 1996; Ganga and Chezhiyan, 2002; Bakry et al., 2007; Goigoux et al., 2013; Salles Pio et al., 2014; Do Amaral et al., 2015). Although there are several reports on induction of polyploidization in banana aiming to generate tetraploid breeding lines towards triploid breeding, most studies are limited to the establishment of *in vitro* polyploidization systems for specific cultivars and isolation of induced tetraploids. There are currently no reports on characterization of induced tetraploids for their use in triploid breeding. Following the creation of induced tetraploids, there is a need for the evaluation of their agronomic/morphological characteristics, fruit quality, and fertility to ascertain their suitability for triploid breeding.

The triploid status, limited fertility, and narrow genetic variability of most cultivated bananas necessitate the use of diploid bananas as a source of diversity for genetic improvement. Wild *M. acuminata* (AA) diploids are quite diverse and have been differentiated into four sub-species (Perrier et al., 2009). Recently, AA wild and cultivated diploids have been further differentiated into several distinct clusters based on cytological and molecular characterization, reflecting evolutionary relationships as well as geographical origins (Christelová et al., 2017). Particularly, diploid cultivars implicated in the evolutionary process of present cultivated varieties may constitute a source of diversity with relevance for banana improvement (Bakry et al., 2009; Brown et al., 2017; Perrier et al., 2018). For example, the AA cultivars of the ssp. *banksii* cluster from Papua New Guinea (PNG), which may have contributed to the A genome in plantain cultivars (Perrier et al., 2009; Hippolyte et al., 2012; Christelová et al., 2017), could be of relevance to plantain breeding. AA diploid cultivars with origins from PNG have also been reported to have high pVAC content with potential for vitamin A biofortification (Fungo and Pillay, 2011; Amah et al., 2019). Such valuable diploid germplasm can serve as progenitors for induced polyploidization to generate tetraploids for triploid breeding. Induced polyploidization is a useful breeding tool for creating novel genetic variation for cultivar improvement and could offer possibilities to utilize existing pVAC diversity within the diploid genepool for biofortification in *Musa* spp.

The aim of this study was to assess the effect of *in vitro* polyploidization on agronomic attributes, pVAC content, and pollen fertility on induced tetraploids derived from diploid banana genotypes. These findings provide a basis for the potential application of polyploid induction as a breeding strategy towards banana biofortification.

## Materials and Methods

### Plant Material

Six diploid AA genotypes (**Table 1**), were subjected to *in vitro* polyploidization to generate tetraploids. *In vitro* polyploidization was carried out as described by Bakry et al. (2007) using oryzalin (PESTANAL^®^, Sigma-Aldrich). Proliferating shoot clusters were established from *in vitro* shoot tips on Murashige and Skoog (MS) basal medium (Sigma-Aldrich) supplemented with 4 mg l^−1^ 6-benzylaminopurine (BAP), 30 g l^−1^ sucrose and 2.0 g l^−1^ Gelrite (Gelzan™, Sigma-Aldrich) with pH adjusted to 5.8 ± 0.1. Selected bud clusters were subjected to oryzalin treatment (45 µM; 15.6 mg l^−1^) for 48 hours on a gyratory shaker at 90 rpm in liquid MS medium, after which they were rinsed in sterile distilled water for 24 hours at 90 rpm. Clusters were transferred to semi-solid MS medium with 1 mg l^−1^ BAP. and surviving plants were sub-cultured at 30-day intervals four times to allow chimera dissociation and plantlet development. Young leaf tissue was obtained from selected, fully developed plantlets for ploidy analysis. Ploidy level of each accession was estimated by ﬂow cytometry according to Dolezel et al. (2007), using a reference AA diploid, Calcutta 4 as external standard.

**Table 1:** Diploid banana genotypes used in this study.

Genotype	Status	Origin*	Cluster**	Induced 4*x* ***
ITC.0266 Sowmuk	Cultivar	Unknown	AAcv *banksii* sensu lato	2 (Sowmuk 1, Sowmuk 2)
ITC.0298 Beram	Cultivar	Indonesia	AAcv IndonTriNG	1 (Beram 1)
ITC.0259 Galeo	Cultivar	Unknown	AAcv IndonTriNG	2 (Galeo 1, Galeo 2)
ITC.0507 Pisang Madu	Cultivar	Unknown	n/a	2 (P Madu 1, P Madu 2)
ITC.0712 AAcv Rose	Cultivar	Indonesia	*M. acuminata* ssp. *malaccensis*	1 (AAcv Rose 1)
25447-S7	Hybrid****	Nigeria	n/a	2 (25447 1, 25447 2)

*Country of origin and classification as stated in MGIS database (www.cropdiversity.org); **Specific cluster as described in Christelová et al. (2017); ***Number and genotype name of isolated tetraploid lines used for further evaluation; ****Pedigree 2829-62 (Bobby Tannap x Calcutta 4) x 9128-3 (Tjau lagada x Pisang lilin).

Induced tetraploids were isolated from separate doubling events for each genotype for further evaluation (**Table 1**). Tetraploids and their diploid progenitors were established on proliferation medium to generate clones, and plantlets were rooted on MS medium devoid of BAP and supplemented with 1 mg l^−1^ 1-naphtaleneacetic acid (NAA), for 40 days, after which they were simultaneously acclimatized in a screenhouse for field establishment.

### Research Site and Experimental Design

Experimental plants were established at the IITA Ibadan research station (3° 54’ E, 7° 30’ N, at 240 m a.s.l.), in the sub humid-derived savanna agro-ecology where the soil type is predominantly a ferric luvisol. The rainfall pattern is bimodal, with two distinct rainy seasons, the first from April to July, and the second from August to November. Fully acclimatized plants of all six diploid progenitors and 10 induced tetraploids (**Table 1**) were planted in a completely randomized design with nine replications. All plants were grown under standard field conditions at a spacing of 3 m × 2 m at a density of 1,666 plants ha^−1^. Plants were evaluated between the months of April 2017 and May 2018.

### Agronomic Assessment

Agronomic characteristics (vegetative and yield-related traits) were evaluated at flowering and at harvest. Plant height (PHT), pseudostem girth at 100 cm from soil surface (PGT) and number of suckers (NSF) were recorded at flowering. Bunch weight (BWT), number of hands or clusters (NH), total number of fingers or fruits (NF), fruit weight (FWT), fruit length (FLT) and fruit circumference (FC) were recorded at harvest when fruits were filled or when a fruit showed signs of yellowing. Fruit measurements were taken from the middle fruit of the third hand. Flowering date was recorded upon emergence of the flag leaf and harvest date was recorded at harvest. Days to flowering (DF) was recorded as the number of days between planting and flowering. Days to fruit maturity (DFM) were recorded as number of days between flowering and harvesting.

### Carotenoid Quantification

Sample preparation, carotenoid extraction, and quantification through high-performance liquid chromatography (HPLC) were carried out as previously described (Amah et al., 2019). Briefly, carotenoids were extracted from finely macerated ripe fruit samples (10 g) using acetone as a solvent and partitioned into petroleum ether *via* a separatory funnel. Petroleum ether extracts were then filtered through anhydrous sodium sulphate, dried under nitrogen gas, and reconstituted in dichloromethane/methanol (v/v) for analysis.

Separation and quantification of individual carotenoids was carried out on a Waters Alliance e2695 HPLC system (Waters Corporation, Milford, MA, USA) equipped with a polymeric YMCTM C30 column (4.6 × 250 mm, 5-µm particle size) and a photodiode array detector. The UV spectrum was observed at 200 to 600 nm, and carotenoids were detected at 450 nm. Data collection and processing were conducted using Empower software (Waters Corporation, Milford, MA, USA). Lutein, α-carotene, *trans*-β-carotene (*trans*-BC), 13-*cis*-β-carotene (13-*cis*-BC), and 9-*cis*-β-carotene (9-*cis*-BC) were identified through an external standard method based on the calibration curve established from pure standards and verification of absorption spectrum and co-elution with authentic commercial standards (β-carotene, α-carotene, and lutein).

Total carotenoids (TC) with provitamin A activity was computed as: pVACs (µg g^−1^ of fresh weight) = α-carotene +13-*cis*-BC + 9-*cis*-BC + *trans*-BC and TC computed as TC (µg g^−1^) = total pVACs + lutein. Provitamin A content expressed in terms of β-carotene equivalents (BCE) was also computed as BCE (µg g^−1^) = 0.5 (α-carotene +13-*cis*-BC + 9-*cis*-BC) + *trans*-BC.

### Pollen Viability Assessment

Pollen viability tests were carried out using 1% 2,3,5-triphenyltetrazolium chloride (TTC) stain diluted in Tris buffer (hydrochloric acid 0.15 M, pH 7.8) as described by Soares et al. (2015). Male flowers were collected from each genotype at anthesis (7.30–10.30 am) and immediately conveyed to the laboratory for analysis. Pollen grains were collected from two anthers of the same flower and manually spread on a glass slide, after which a drop of TTC stain was added. The preparation was covered with a cover slip and allowed to stand for two hours to allow uptake of the stain. Two slides were prepared of each genotype (four flowers from each individual plant) and preparations were observed under bright field illumination using a light microscope (Olympus BX51). Pollen grains stained with TTC appears light pink or red when viable and remains transparent when non-viable. Viable and non-viable pollen grains were recorded from observation of three selected microscopic fields of each slide. The percentage of stained pollen grain was calculated from the pollen counts and expressed as percentage pollen viability.

### Statistical Analysis

All data were collected, summarized, and analyzed using SAS software version 9.4 for Windows Copyright ^©^ 2002–2015 by SAS Institute Inc., Cary, NC, USA. The PROC GLM statement was used for one-way ANOVA followed by Student-Newman-Keuls test and Duncan’s Multiple Range Test to detect significant differences among means for agronomic and carotenoid traits, respectively, and the statistical significance level was set to 0.05. Heatmap correlation (genotypes versus variables) was performed using “heatmap.2” function through gplots v3.0.1.1 library implemented in R (R Core Team, 2018).

## Results

### Agronomic Characteristics

**Figure 1:**
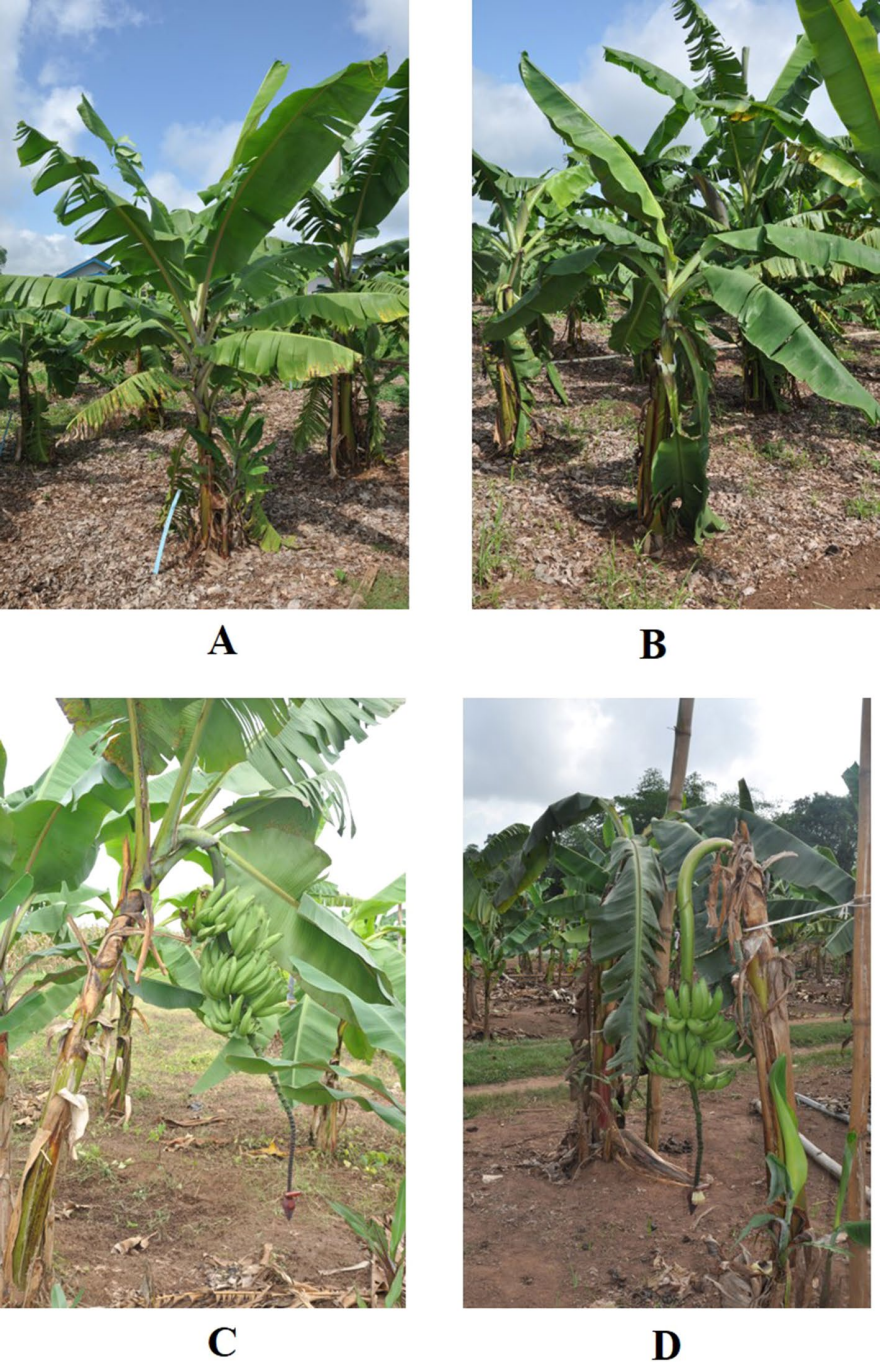
Leaf characteristics of diploid and induced tetraploid banana cultivar Galeo. Pre-flowered plants of diploid **(A)** and induced tetraploid **(B)** and mature plants for diploid **(C)** and induced tetraploid **(D)** of cultivar Galeo.

**Figure 2:**
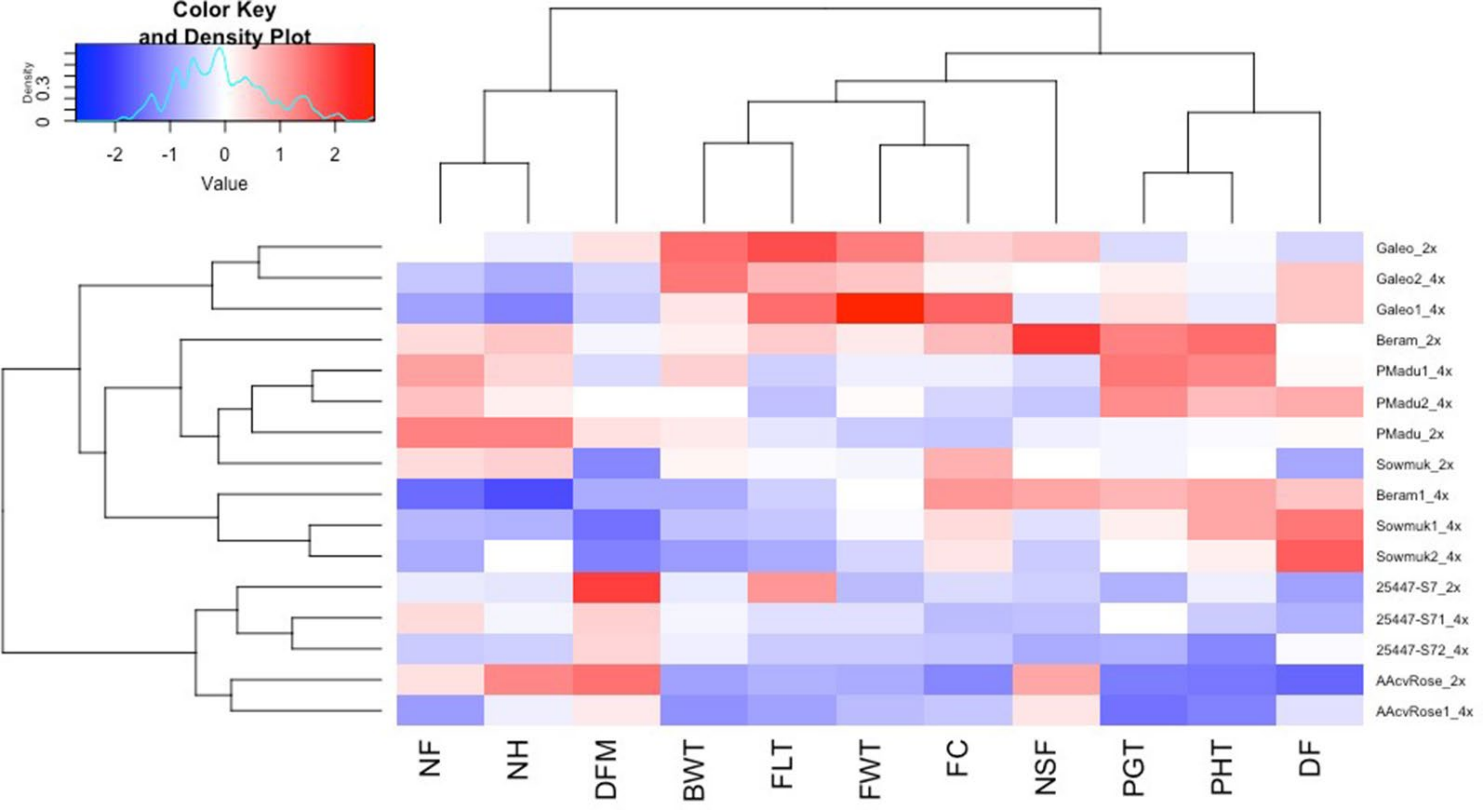
Heatmap and hierarchical clustering for agronomic traits in diploid and induced tetraploid bananas. Genotype names on the vertical axis followed by 2x and 4x indicates diploids and tetraploid lines respectively. DF, days to flowering; PHT, plant height; PGT, Plant girth; NSF, number of suckers at flowering; DFM, days to fruit maturity; BWT, bunch weight; NH, number of hands; NF, number of fruits; FLT, fruit length; FC, fruit circumference; FWT fruit weight. Bright blue indicates lowest values while bright red indicates highest values for each trait.

Results for agronomic characteristics are only presented for plants harvested at full maturity, as some plants suffered wind damage. Significant phenotypic differences were observed between diploid plants and their induced tetraploids for vegetative and yield traits. Tetraploid plants had longer, curved, and drooping leaves, while diploid plants had shorter and more erect leaves (**Figure 1**). Data for vegetative characteristics are presented in **Table 2** and illustrated in **Figure 2**. Generally, number of days to flowering (384.04) was significantly longer for induced tetraploids plants than for diploids (299.71) but the fruit maturity time was significantly shorter for induced tetraploids (81.21) than for original diploids (101.55). Plant height and plant girth did not differ significantly for both groups but the number of suckers at flowering was significantly higher for diploids (7.34) than for induced tetraploids (3.87).

**Table 2:** Vegetative characteristics of diploid and induced tetraploid banana lines.

Genotype	Ploidy	n	Vegetative characteristics (mean ± standard deviation)				
			DF	PHT (cm)	PGT (cm)	NSF	DFM
Sowmuk	2*x*	6	272.00 ± 41.53^ef^	231.33 ± 19.61^bcd^	36.83 ± 4.31^de^	5.50 ± 1.87^bcde^	53.50 ± 2.66^g^
Sowmuk 1	4*x*	5	460.40 ± 60.87^ba^	270.00 ± 9.35^ab^	39.60 ± 4.88^cde^	4.00 ± 1.22^bcde^	48.60 ± 2.07^g^
Sowmuk 2	4*x*	5	481.80 ± 39.77^a^	238.00 ± 8.37^bcd^	37.40 ± 2.19^de^	3.20 ± 1.92^de^	51.80 ± 6.53^g^
Beram	2*x*	6	343.33 ± 25.07^cde^	296.67 ± 41.19^a^	49.17 ± 9.24^ab^	13.50 ± 6.35^a^	87.17 ± 28.48^cdef^
Beram 1	4*x*	3	395.33 ± 76.79^bcd^	271.67 ± 55.08^ab^	44.33 ± 13.05^abcd^	9.00 ± 4.36^bc^	65.33 ± 6.66^fg^
Galeo	2*x*	8	309.13 ± 32.38^de^	226.25 ± 8.35^bcd^	34.50 ± 5.58^def^	8.00 ± 2.33^bcd^	101.00 ± 2.98^cd^
Galeo 1	4*x*	4	395.75 ± 73.22^bcd^	220.00 ± 20.41^cd^	40.75 ± 2.87^bcde^	4.25 ± 1.26^bcde^	74.75 ± 23.95^ef^
Galeo 2	4*x*	3	394.67 ± 19.09^bcd^	223.33 ± 17.56^cd^	39.67 ± 5.86^cde^	5.33 ± 2.52^bcde^	77.67 ± 8.74d^ef^
P Madu	2*x*	8	351.13 ± 49.92^cde^	226.88 ± 19.81^bcd^	36.88 ± 3.23^de^	4.75 ± 1.04^bcde^	100.75 ± 8.24^cd^
P Madu 1	4*x*	4	353.25 ± 10.72^cde^	283.75 ± 14.93^a^	50.00 ± 2.58^a^	3.75 ± 0.50^cde^	79.50 ± 15.72^def^
P Madu 2	4*x*	7	415.86 ± 50.94^abc^	261.43 ± 22.86^abc^	48.14 ± ± 1.95^abc^	3.00 ± 1.53^de^	89.14 ± 3.63^cde^
AAcv Rose	2*x*	5	217.00 ± 42.77^f^	170.00 ± 7.91^e^	26.20 ± 2.86^fg^	9.20 ± 4.38^b^	132.20 ± 7.40^b^
AAcv Rose 1	4*x*	3	319.33 ± 20.50^de^	173.33 ± 17.56^e^	25.33 ± 4.51^g^	6.67 ± 0.58^bcde^	97.00 ± 6.56^cde^
25447-S7	2*x*	5	266.00 ± 58.61^ef^	221.00 ± 42.92^cd^	30.60 ± 5.37^efg^	3.40 ± 1.52^de^	148.00 ± 16.19^a^
25447-S7 1	4*x*	5	278.20 ± 27.19^ef^	205.00 ± 20.31^de^	37.60 ± 4.51^de^	2.80 ± 1.48^de^	105.40 ± 10.48^c^
25447-S7 2	4*x*	8	339.13 ± 39.70^cde^	175.63 ± 19.35^e^	30.63 ± 3.25^efg^	2.00 ± 0.76^e^	103.38 ± 9.91^c^
All 2*x*		38	299.71 ± 60.49^B^	230.21 ± 42.40^A^	36.08 ± 8.47^A^	7.34 ± 4.50^A^	101.55 ± 31.40^A^
All 4*x*		47	384.04 ± 74.35^A^	230.21 ± 43.29^A^	39.28 ± 8.21^A^	3.87 ± 2.36^B^	81.21 ± 22.28^B^
All genotypes		85	346.34 ± 80.11	230.21 ± 42.64	37.85 ± 8.43	5.42 ± 3.87	90.31 ± 28.46

n, number of plants sampled; DF, days to flowering, PHT, plant height; PGT, Plant girth; NSF, number of suckers at flowering; DFM, days to fruit maturity; means followed by the same case letter within a column are not significantly different at P < 0.05.

**Figure 3:**
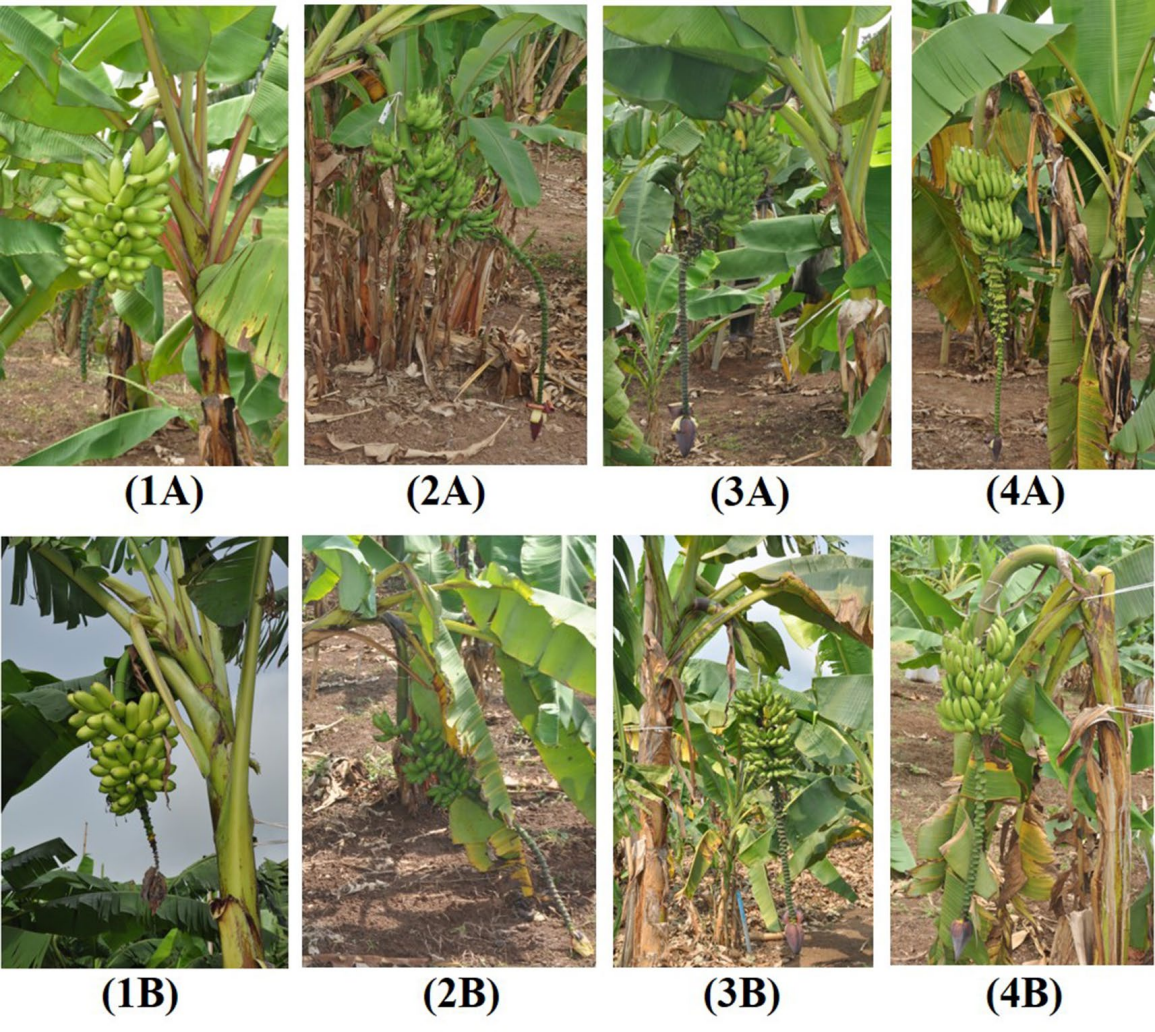
Bunch characteristics of diploid and induced tetraploid bananas. 2x Sowmuk **(1A)**; 4*x* Sowmuk **(1B)**; 2*x* AAcv Rose **(2A)**; 4*x* AAcv Rose **(2B)**; 2*x* Pisang Madu **(3A)**; 4*x* Pisang Madu **(3B)**; 2*x* 25447-S7 **(4A)**; 4*x* 25447-S7 **(4B)**.

Induced tetraploids generally had significantly lower bunch weights (6.01 kg) than diploids (7.76 kg) but variable changes were observed for individual genotypes (**Figure 3**; **Table 3**). Notably, tetraploids from the hybrid 25447-S7 (25447-S7 1 and 2) and Pisang Madu (P Madu 1) had slightly higher bunch weights than their diploid counterparts, while Sowmuk 2 recorded the highest reduction in bunch weight (> 50%) following polyploidization which was significantly different from the diploid version, though these changes in bunch weights were generally not significant (**Figure 3**, **Table 3**). Similarly, the number of hands per bunch was higher (7.39) for diploids than for induced tetraploids (6.00), but this difference was only significant for Beram and AAcv Rose (**Table 3**). Mean number of fruits was significantly higher for diploids (107.63) than tetraploids (82.43) but mean fruit weight and circumference were variable among doubled and non-doubled genotypes.

**Table 3:** Yield attributes and pollen viability of diploid and induced tetraploid banana lines.

Genotype	Ploidy	n	Yield - related traits (mean ± standard deviation)						Pollen viability (% )
			BWT (kg)	NH	NF	FLT (cm)	FC (cm)	FWT (cm)	
Sowmuk	2*x*	6	7.25 ± 2.72^abc^	7.33 ± 1.37^abcd^	107.83 ± 42.37^abcd^	14.67 ± 1.21^d^	11.50 ± 1.05^abc^	55.33 ± 19.36^cd^	
Sowmuk 1	4*x*	5	4.20 ± 2.17^bc^	5.40 ± 0.55^def^	67.20 ± 7.60^de^	12.60 ± 1.34^d^	10.80 ± 1.64^abc^	58.80 ± 22.99^cd^	
Sowmuk 2	4*x*	5	2.80 ± 1.48^bc^	6.60 ± 0.55^bcde^	63.80 ± 10.64^de^	11.40 ± 0.89^d^	10.60 ± 1.14^abc^	42.80 ± 8.35^cd^	
Beram	2*x*	6	7.50 ± 2.95^abc^	7.50 ± 1.22^abc^	107.17 ± 20.64^abcd^	17.17 ± 1.72^c^	11.33 ± 0.52^abc^	71.50 ± 14.18^cd^	
Beram 1	4*x*	3	3.50 ± 0.50^bc^	4.00 ± 1.00^f^	41.67 ± 23.67^e^	13.00 ± 0.00^d^	12.00 ± 1.73^ab^	61.33 ± 6.35^cd^	
Galeo	2*x*	8	12.56 ± 2.44^a^	6.38 ± 0.74^cde^	92.00 ± 8.28^bcd^	22.50 ± 1.31^a^	11.00 ± 0.53^abc^	115.63 ± 39.42^b^	82.12 ± 5.12^b^
Galeo 1	4*x*	4	8.00 ± 4.32^abc^	4.75 ± 0.50^ef^	59.50 ± 4.80^de^	21.00 ± 1.83^ab^	13.00 ± 0.82^a^	168.75 ± 18.84^a^	76.54 ± 1.97^cd^
Galeo 2	4*x*	3	12.33 ± 8.50^a^	5.33 ± 0.58^def^	72.67 ± 5.77^cde^	18.00 ± 3.46^c^	10.33 ± 2.08^bcd^	87.00 ± 35.93^bc^	75.80 ± 2.26^cd^
P Madu	2*x*	8	7.75 ± 1.83^abc^	8.50 ± 1.07^a^	138.25 ± 42.24^a^	13.75 ± 1.39^d^	9.00 ± 1.31^cd^	38.00 ± 11.14^d^	73.98 ± 5.48^de^
P Madu 1	4*x*	4	8.75 ± 2.63^ab^	7.25 ± 0.50^abcd^	127.25 ± 8.88^ab^	13.00 ± 1.41^d^	9.75 ± 0.50^bcd^	52.75 ± 5.91^cd^	78.26 ± 4.80^c^
P Madu 2	4x	7	6.71 ± 1.11^bc^	6.86 ± 1.07^abcd^	116.86 ± 18.73^ab^	12.29 ± 1.60^d^	9.29 ± 1.25^bcd^	64.43 ± 40.8^cd^	72.17 ± 10.21^ef^
AAcv Rose	2*x*	5	3.00 ± 0.71^bc^	8.40 ± 0.55^ab^	105.40 ± 2.07^abcd^	11.60 ± 0.89^d^	7.80 ± 0.45^d^	26.40 ± 4.39^d^	86.81 ± 5.99^a^
AAcv Rose 1	4*x*	3	2.33 ± 0.58^c^	6.33 ± 1.53^cde^	58.33 ± 16.17^de^	11.00 ± 1.00^d^	9.00 ± 1.00^cd^	32.00 ± 7.00^d^	82.22 ± 7.64^b^
25447-S7	2*x*	5	5.80 ± 2.17^bc^	6.20 ± 1.10^cde^	86.20 ± 30.59^bcde^	19.40 ± 5.03^bc^	9.40 ± 3.21^bcd^	31.60 ± 20.28^d^	73.68 ± 4.39^def^
25447-S7 1	4*x*	5	6.20 ± 2.28^bc^	6.40 ± 0.89^cde^	106.40 ± 19.03^abcd^	13.60 ± 0.55^d^	8.80 ± 0.45^cd^	48.00 ± 11.47^cd^	78.10 ± 4.97^c^
25447-S7 2	4*x*	8	6.00 ± 3.42^bc^	5.88 ± 0.99^cde^	75.50 ± 15.93^cde^	12.75 ± 1.04^d^	9.00 ± 1.07^cd^	38.38 ± 14.73^d^	73.24 ± 8.73^def^
Calcutta 4	2x								70.51 ± 10.27^f^
All 2*x*		38	7.76 ± 3.62^A^	7.39 ± 1.33^A^	107.63 ± 32.72^A^	16.74 ± 4.30^A^	10.08 ± 1.87^A^	60.00 ± 38.75^A^	
All 4*x*		47	6.01 ± 3.78^B^	6.00 ± 1.18^B^	82.43 ± 29.35^B^	13.57 ± 3.03^B^	10.06 ± 1.67^A^	62.40 ± 40.71^A^	
All genotypes		85	6.79 ± 3.79	6.62 ± 1.42	93.69 ± 33.20	14.99 ± 3.96	10.07 ± 1.75	61.33 ± 39.63	

n, number of plants sampled; BWT, bunch weight; NH, number of hands; NF, number of fruits; FLT, fruit length; FC, fruit circumference; FWT, fruit weight; means followed by the same case letter within a column are not significantly different at p < 0.05.

Hierarchical clustering and heat maps of agronomic characteristics of all 16 diploid and induced tetraploid lines were plotted to illustrate the influence of doubling on the variables measured (**Figure 2**). All entries were clustered in to five main groups based on the 11 agronomic traits measured. The diploid Galeo and its tetraploid forms Galeo 1 and Galeo 2 grouped together in a single cluster showing higher values for bunch weight, fruit length, fruit weight, and fruit circumference, than all other entries. Tetraploids generally had higher days to flowering than diploids and three tetraploids (Beram 1, Sowmuk 1, and Sowmuk 2) with higher days to flowering clustered together. With regard to traits, 11 agronomic traits were grouped into three different groups with plant girth, plant height, and days to flowering clustering together in a first group; bunch weight, fruit length, fruit weight, fruit circumference, and number of suckers at flowering clustering together in a second group and number of fruits, number of hands and days to fruit maturity clustered together in a third group.

### Carotenoid Content

**Figure 4:**
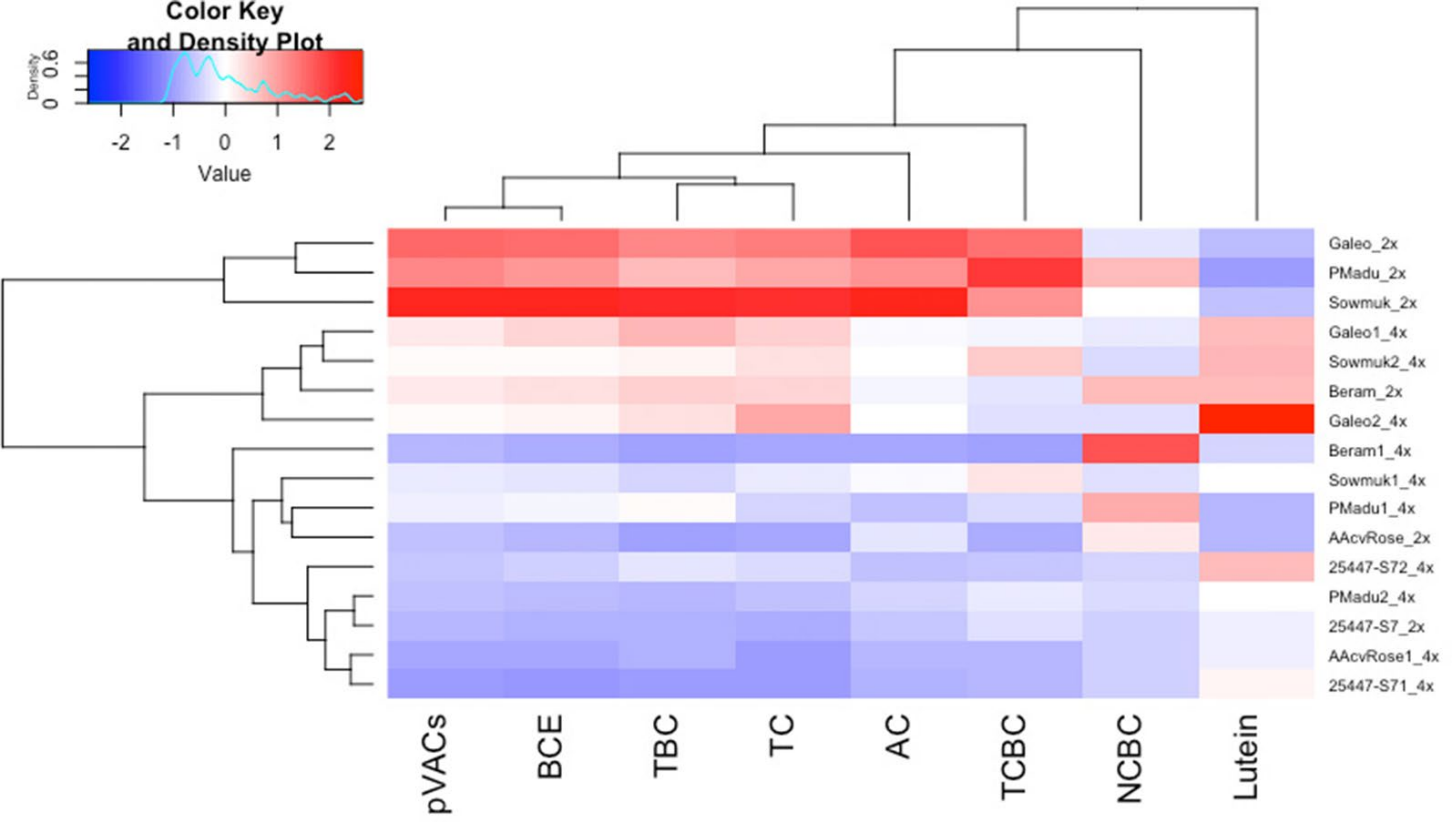
Heatmap and hierarchical clustering for carotenoid traits in diploid and induced tetraploid bananas. Genotype names followed by 2x and 4x indicates diploids and tetraploid lines respectively. AC, α-carotene; TCBC, 13-*cis*-β-carotene; NCBC, 9-*cis* β-carotene; TBC, *trans*-β-carotene; BCE, β-carotene equivalents; pVACs, total carotenoids with vitamin A activity; TC HPLC, total carotenoids determined by HPLC. Bright blue indicates lowest values while bright red indicates highest values for each trait.

Data for carotenoid traits are presented in **Table 4** and illustrated in **Figure 4**. The predominant carotenoids isolated were pVACs α-carotene, β-carotene (*cis* and *trans* versions) and lutein. Total carotenoids determined from HPLC (µg g^−1^ FW) was highest for diploid Sowmuk (8.58) and Galeo (6.80) and lowest for AAcv Rose (1.50) and 25447-S7 (1.66). Generally, lutein content was higher in tetraploids (1.09 µg g^−1^ FW) than in diploids (0.54 µg g^−1^ FW) while all other carotenoids were higher in diploids than in tetraploids except for 25447-S7. Individual genotypes showed increase in lutein content after doubling, but this was only significant for Sowmuk and Galeo, which had the highest carotenoid content at the diploid and tetraploid level. In contrast, induced tetraploids for the genotypes Sowmuk, Beram, Galeo, and P Madu had significantly lower pVACs α-carotene and *trans* β-carotene with consequently lower BCE and TC content (**Table 4**). *Cis* carotenes 13-*cis* β-carotene and 9-*cis* β-carotene decreased with doubling in all genotypes, but the decrease was not significant. Hierarchical clustering and heat maps (**Figure 4**) depicted 3 main clusters for entries based on carotenoid traits measured. The first main cluster contained three (Galeo, P Madu, and Sowmuk) of the five diploids sampled, which were characterized by high values for all carotenoid traits except lutein. The second cluster contained three tetraploids (Galeo 1, Sowmuk 2 and Galeo 2) which showed moderately lower values for the carotenoid traits except for lutein. The third cluster had the highest number of genotypes including 7 of the 10 tetraploids sampled. Genotypes in this cluster were characterized by low carotenoid values. In general, the heatmap shows a decline in carotenoid values of tetraploids when compared to diploids. With regard to clustering of traits, 9-*cis*-BC and lutein were distinct from the rest of the carotenoid traits measured.

**Table 4:** Carotenoid content of diploid and induced tetraploid banana lines.

Genotype	Ploidy	Carotenoid content (Mean µg g^−1^ ± standard deviation)							
		Lutein	α-carotene	13-*cis*-β-carotene	9-*cis* β-carotene	*trans*-β-carotene	BCE	pVACs	TC HPLC
Sowmuk	2*x*	0.36 ± 0.38^cde^	3.76 ± 0.54^a^	0.64 ± 0.22^abc^	0.17 ± 0.04^ab^	3.65 ± 1.09^a^	5.94 ± ± 1.16^a^	8.22 ± 1.32^a^	8.58 ± 1.54^a^
Sowmuk 1	4*x*	0.88 ± 0.58b^cde^	0.99 ± 0.23^cde^	0.40 ± 0.25^cde^	0.07 ± 0.03^b^	0.74 ± 0.37^efg^	1.46 ± 0.24^efg^	2.19 ± 0.28^cd^	3.07 ± 0.37^de^
Sowmuk 2	4*x*	1.45 ± 1.67^b^	1.08 ± 0.98^cd^	0.47 ± 0.39^bcd^	0.05 ± 0.02^b^	1.37 ± 0.49^cde^	2.17 ± 0.18^cde^	2.96 ± 0.86^c^	4.41 ± 2.52^cd^
Beram	2*x*	1.41 ± 0.13^bc^	0.93 ± 0.38^cdef^	0.21 ± 0.1^def^	0.42 ± 0.61^ab^	1.77 ± 0.55^bcd^	2.54 ± 0.66^cd^	3.32 ± 0.83^c^	4.74 ± 0.76^c^
Beram 1	4*x*	0.54 ± 0.68^bcde^	0.08 ± 0.04^f^	0.01 ± 0.00^f^	0.78 ± 1.08^a^	0.13 ± 0.16^g^	0.56 ± 0.69^gh^	0.99 ± 1.21d^e^	1.53 ± 0.53^ef^
Galeo	2*x*	0.34 ± 0.32^de^	3.07 ± 0.63^ab^	0.74 ± 0.32^ab^	0.08 ± 0.02^b^	2.57 ± 0.16^b^	4.52 ± 0.33^b^	6.47 ± 0.52^b^	6.80 ± 0.73^b^
Galeo 1	4*x*	1.42 ± 0.89^bc^	0.99 ± 0.57^cde^	0.26 ± 0.14^def^	0.09 ± 0.04^b^	2.06 ± 0.75^bc^	2.73 ± 1.04^c^	3.40 ± 1.34^c^	4.83 ± 0.89^c^
Galeo 2	*4x*	2.87 ± 1.35^a^	1.14 ± 0.21^c^	0.20 ± 0.07^def^	0.07 ± 0.05^b^	1.56 ± 0.88^cde^	2.26 ± 0.98^cd^	2.97 ± 1.07^c^	5.84 ± 0.28^bc^
P Madu	2*x*	0.09 ± 0.02^e^	2.41 ± 0.83^b^	0.92 ± 0.31^a^	0.41 ± 0.56^ab^	1.97 ± 0.74^bc^	3.83 ± 0.41^b^	5.70 ± 1.10^b^	5.79 ± 1.08^bc^
P Madu 1	4*x*	0.27 ± 0.09^e^	0.35 ± 0.08^cdef^	0.19 ± 0.03^def^	0.47 ± 0.56^ab^	1.29 ± 0.05^cdef^	1.79 ± 0.36^def^	2.29 ± 0.69^cd^	2.57 ± 0.77^ef^
P Madu 2	4*x*	0.89 ± 0.13^bcde^	0.58 ± 0.18^bcde^	0.24 ± 0.20^def^	0.05 ± 0.03^b^	0.37 ± 0.09^fg^	0.80 ± 0.18^fgh^	1.23 ± 0.34^de^	2.11 ± 0.22^ef^
AAcv Rose	2*x*	0.28 ± 0.17^e^	0.79 ± 0.38^cdef^	0.04 ± 0.02^ef^	0.25 ± 0.06^b^	0.15 ± 0.05^g^	0.68 ± 0.11^gh^	1.22 ± 0.26^de^	1.50 ± 0.15^ef^
AAcv Rose 1	4*x*	0.71 ± 0.06^bcde^	0.23 ± 0.03^def^	0.08 ± 0.01^ef^	0.01 ± 0.00^ab^	0.28 ± 0.04^g^	0.43 ± 0.05^gh^	0.59 ± 0.06^e^	1.30 ± 0.10^f^
25447-S7	2*x*	0.74 ± 0.14^bcde^	0.43 ± 0.03^cdef^	0.21 ± 0.10^def^	0.01 ± 0.00^b^	0.28 ± 0.04^g^	0.60 ± 0.03^gh^	0.93 ± 0.06^de^	1.66 ± 0.19^ef^
25447-S7 1	4*x*	0.98 ± 0.11^bcde^	0.16 ± 0.00^ef^	0.08 ± 0.04^ef^	0.01 ± 0.00^b^	0.06 ± 0.01^g^	0.18 ± 0.02h	0.30 ± 0.04^e^	1.28 ± 0.11^f^
25447-S7 2	4*x*	1.40 ± 0.18^bcd^	0.35 ± 0.11^cdef^	0.12 ± 0.05^def^	0.02 ± 0.02^b^	0.86 ± 0.64^defg^	1.10 ± 0.72^efgh^	1.35 ± 0.79^de^	2.76 ± 0.84^ef^
All 2*x* (n = 18)		0.54 ± 0.49^B^	1.90 ± 1.36^A^	0.46 ± 0.38^A^	0.22 ± 0.33^A^	1.73 ± 1.36^A^	3.02 ± 2.07^A^	4.31 ± 2.87^A^	4.84 ± 2.76^A^
All 4*x* (n = 27)		1.09 ± 0.83^A^	0.58 ± 0.47^B^	0.2 ± 0.18^B^	0.14 ± 0.35^A^	0.85 ± 0.75^B^	1.31 ± 0.95^B^	1.77 ± 1.21^B^	2.86 ± 1.59^B^
mean		0.87 ± 0.76	1.11 ± 1.13	0.3 ± 0.30	0.17 ± 0.34	1.20 ± 1.11	2.00 ± 1.70	2.79 ± 2.37	3.66 ± 2.32

BCE, β-carotene equivalents; pVACs, total carotenoids with vitamin A activity; TC HPLC, total carotenoids determined by HPLC; means were calculated from three plants sampled for each genotype; means followed by the same case letter within a column are not significantly different at P < 0.05.

### Pollen Viability

**Figure 5:**
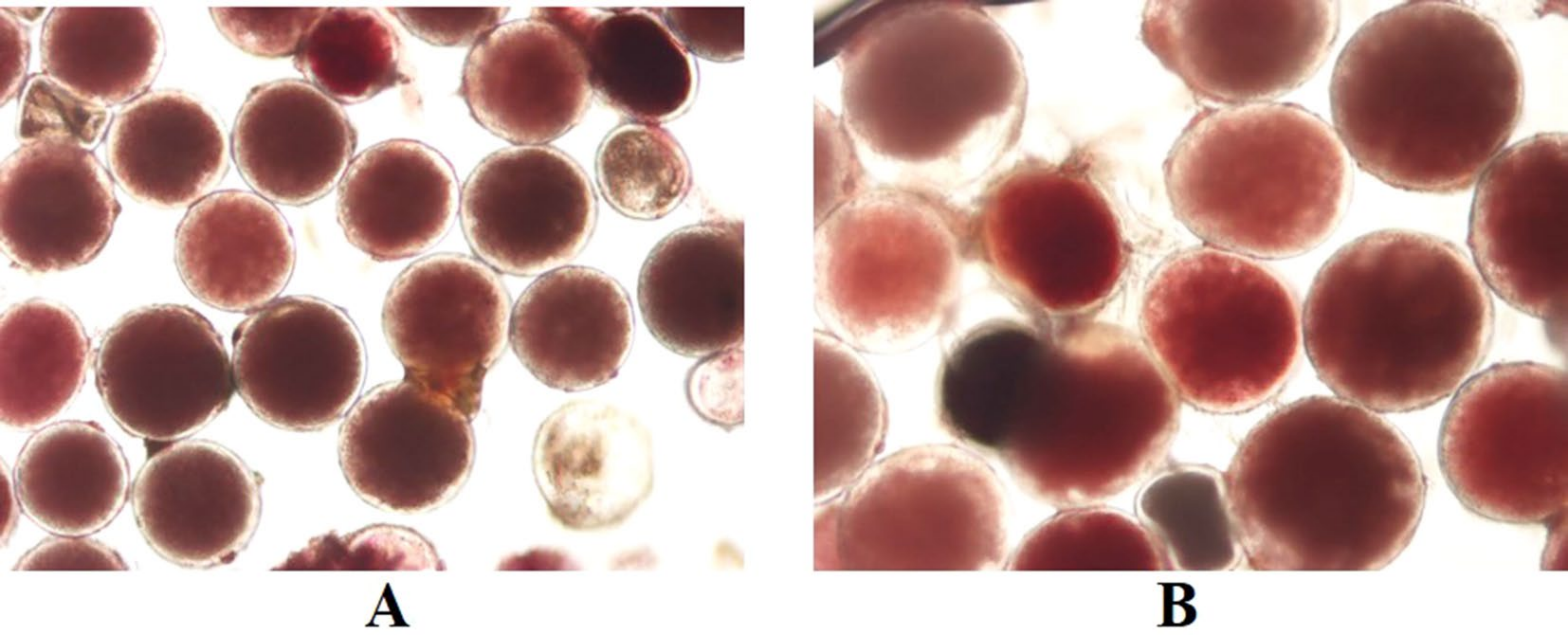
TTC stained pollen from diploid **(A)** and tetraploid **(B)** banana cultivar Galeo, showing high viability. Magnification 200x.

Pollen viability *via* staining with TTC was used to estimate male fertility for four diploid genotypes and six induced tetraploids (**Figure 5** and **Table 3**). Genotypes Sowmuk and Beram and their corresponding induced tetraploids were not included in the viability assessment because they recorded very low pollen counts (<10 per field) and exhibited degenerated male buds (**Figures 3:1A, 1B**). Pollen viability ranged from 70.51% to 86.81% with a mean of 76.95%. All genotypes tested had significantly higher viability than the Calcutta 4 diploid control. For genotypes Galeo and AAcv Rose, the pollen viability of the diploid was significantly higher than that of the induced tetraploids. Genotypes 25447-S7 and P Madu, recorded induced tetraploids with significantly higher viability than the diploid control (**Table 3**).

## Discussion

Polyploidy is believed to impart phenotypic changes such as increased cell nuclei and size, leading to increased plant organs (gigas effect) as a consequence of gene duplications (Hegarty et al., 2013; Sattlser et al., 2016). Other resulting physiological or phenological characteristics may include larger leaves, greater vigor and biomass, increased resistance to biotic and abiotic stress, higher content of chemical substances, altered flowering time, increased fertility, and better post-harvest quality (Dhooghe et al., 2011). Although there are several reports on polyploidy induction in bananas, most reports have focused on developing *in vitro* polyploidization protocols without a comprehensive report on field evaluation of induced tetraploids. Thus, in this study, tetraploids obtained from six diploid genotypes were characterized for their agronomic attributes, pVAC contents, and fertility, in comparison with their diploid progenitors.

From field observations, induced tetraploids showed distinct morphological characteristics when compared to the diploid progenitors. Induced tetraploids were characterized by long curved leaves with weak petioles resulting in a drooping leaf habit or fallen/broken leaves, as previously observed in banana (Van Duren et al., 1996; Bakry et al., 2007; Do Amaral et al., 2015). Increased leaf size following polyploidization as a result of cell division and expansion has also been reported in several other plant species, with wide applications in the improvement of leaf size and flowers of ornamental plants (Huang et al., 2015; Tamayo-Ordóñez et al., 2016; Denaeghel et al., 2018; Parsons et al., 2019).

With regard to phenology (**Table 2**, **Figure 2**), longer days to flowering (28%) were observed in induced tetraploids than original diploids, but once plants had flowered, tetraploids matured faster (25%) than diploids. Plant height and girth at flowering was not significantly affected by doubling, but 47% less suckering was observed in induced tetraploids than in diploid plants. This was in contrast to observations by Do Amaral et al. (2015), who reported that plant height and pseudostem diameter increased while number of suckers decreased with polyploidization in Pisang Lilin. Variable changes in yield attributes following polyploidization, were also recorded. Although tetraploids had larger fruits, this did not necessarily result in increased bunch weight because of the associated decrease in number of hands, number of fruits and fruit length. Faster maturity in tetraploids could also be attributable to the fact that they had fewer fruits but the relationship between both traits is still unclear. Nyine et al. (2017) did not find significant correlation between both traits in bananas when a population consisting of 307 genotypes that included diploid, triploid, and tetraploid plants was assessed. Do Amaral et al. (2015) also observed fewer fruits in the tetraploid Pisang Lilin in the first cycle of evaluation, which later increased in the second cycle. Denaeghel et al. (2018) also reported variable effects of polyploidization in the phenotypes of Escallonia. Polyploidization is known to have played a key role in plant evolution and speciation, leading to better adaptation. While it is expected that induced polyploidization would result in superior traits, this varies among crop species. For example, more vigorous plants with larger flowers were observed in the tetraploid Snowdrop windflower (Zahumenická et al., 2018) while a decreased growth rate or plant vigor was observed in tetraploid apples (Xue et al., 2017). Generally, polyploidy is induced *in vitro via* the use of toxic anti-tubulin compounds such as oryzalin, which prevent microtubule formation and inhibits mitosis during metaphase, affecting plant growth and vigor. Podwyszyńska et al. (2015) reported that induced tetraploids of daylily exhibited poorer growth than original diploids in the first year but became more vigorous in the second year, possibly attributable to residual toxic effects of anti-mitotic agents.

Carotenoids are widely distributed in nature and specifically pVACs present in edible plant parts are important for human health. Fruit pVAC content is an important factor to consider for banana biofortification. The effect of polyploidy on compositional traits have been studied in some plant species, indicating increased or decreased concentrations (Trojak-Goluch and Skomra, 2013; Zahumenická et al., 2018). However, studies specific to carotenoids are limited. Carotenoids often present in banana fruit pulp are mainly the pVACs α-carotene and β-carotene (*cis* and *trans* versions) with smaller quantities of lutein (Davey et al., 2009). Using HPLC, the carotenoid content of banana fruit pulp of tetraploids and diploid plants were assessed to determine the effect of doubling. Polyploidization led to a significant decrease in pVACs in induced tetraploids when compared to diploid progenitors, which was associated with a corresponding increase in contents of the non-pVAC lutein (**Figure 4**). Trojak-Goluch and Skomra (2013) also reported significantly lower contents of essential oils in tetraploid hops in comparison to their diploid counterparts. On the other hand, Sanwal et al. (2010) reported higher gingerol content and antioxidant activity in tetraploid clones of ginger than that of the original diploids. Parsons et al. (2019) also observed significant changes in secondary metabolites with a 9% increase in cannabidol from tetraploid *Cannabis sativa* in comparison to their diploid progenitors. The expectation is that whole genome duplication may lead to increased gene expression from increased gene dosage, resulting in enhanced contents of compositional traits like carotenoids. However, this is not always the case as polyploidization may also lead to genome alterations affecting the expression of key genes implicated in hormonal regulation of plant developmental pathways (Sanglard et al., 2017; Xue et al., 2017). Polyploidization may also result in a changed post-transcriptional regulation and translational modification changes of proteins, which play a role in plant biological processes (Wang et al., 2017). Such alterations, possibly leading to the expression of deleterious alleles, may also be associated with poor field establishment and productivity of doubled lines.

The ability to generate progeny depends on gamete fertility, which is known to vary among genotypes. Understanding the pollen viability as an indicator of male fertility or seed set as an indicator of female fertility is critical to ascertain the potential for use of induced tetraploids in hybridizations. The absence of pollen in diploid and tetraploid Sowmuk and Beram (not included in pollen viability studies) is linked to the rapid degeneration of the male bud before maturity. While some diploids may be infertile, fertility may be restored at the tetraploid level. Bakry et al. (2009) reported male and female fertility in tetraploids induced from interspecific AB clones, which were otherwise sterile at the diploid level. In this study, it was observed that diploid and tetraploid versions of genotypes tested had high pollen viability (>70%), which was indeed higher than that of the male fertile diploid Calcutta 4. Notwithstanding, the tetraploid Galeo and AAcv Rose recorded lower pollen viability than their original diploids. However, for P Madu and 25447-S7, pollen viability of tetraploid versions were comparable to, or higher than that of their original diploids. The pollen viability values for induced tetraploids were higher than the 31 to 62.6% viability observed by Soares et al. (2016) on 12 banana tetraploid hybrids assessed for pollen viability using TTC staining, which is a fast and reliable method of accessing pollen viability. Goigoux et al. (2013) also detected viable pollen in doubled diploid Mlali clones Chicame 4*x* and Paka 4*x*, which was not significantly different from the diploids, indicating their potential as male parents in banana breeding. The pollen of tetraploid plants generally appeared larger than pollen of diploid plants (**Figure 5**) possibly also attributable to cell expansion following tetraploidization.

The prevailing ploidy levels in banana include diploids, triploids, and tetraploids, but triploids are believed to be the optimum ploidy level, since they exhibit better agronomic characteristics and produce non-functional gametes, which ensure seedlessness and edibility. In the banana breeding scheme, tetraploids are synthesized from 3*x*–2*x* crosses, and are further crossed with male diploid plants to generate triploids. Therefore, the goal for induced polyploidization in banana breeding is to directly synthesize tetraploids which can be crossed with elite diploids to generate triploids, while keeping the maximum genetic constitution of the diploid at the tetraploid level. Bakry et al. (2009) crossed induced tetraploid clones from Guyod, Galeo, IDN110, and Tjau Lagada with Calcutta 4 and obtained 347 hybrids, which were predominantly (98%) triploids. Oselebe et al. (2010) also pointed out the predominance of triploids in progenies from 4*x*–2*x* crosses. Nukaya et al. (2019) also reported the use of induced autotetraploid kumquats (*Fortunella* spp.) to generate seedless triploids through 4*x*–2*x* crosses and reciprocals. Although induced tetraploids generally showed inferior agronomic and pVAC traits in comparison to original diploids, it is expected that these tetraploids will constitute novel diversity for the generation of triploids with superior traits when crossed with elite diploids through transgressive segregation. However, it will be necessary to further evaluate hybrids from crosses with induced tetraploids to ascertain that traits of interests are ultimately expressed in the triploid progeny. Moreover, once tetraploid lines are produced, other desirable traits such as disease and pest resistance may be introgressed by incorporating different diploid cultivars and hybrids in the breeding scheme.

## Conclusions

Biofortification through conventional breeding is becoming popular as a trusted approach to tackle micronutrient deficiency and breeding programs are utilizing available diversity to enhance micronutrient levels in staple crops. Although considerable diversity exists for pVACs in *Musa* genetic resources, genetic enhancement for pVAC content is still limited owing to several challenges with current breeding strategies. The use of *in vitro* polyploidization was explored as a breeding tool for banana improvement and the effect of polyploidization on agronomic characteristics and pVACs content of induced tetraploids was determined. Induced tetraploid plants showed distinct morphological characteristics, but generally had inferior agronomic characteristics and pVACs when compared to the original diploids. Nevertheless, preliminary fertility assessments indicated that induced tetraploids generated were pollen fertile, hence could provide a significant breeding pool for the synthesis of high pVAC triploid hybrids. While the seemingly negative effect of polyploidization on induced tetraploid is worth noting, polyploidization of diploids offers an opportunity for utilizing useful variability in diploid bananas which may otherwise remain inaccessible for breeding. However, further evaluations are needed to ascertain the (breeding) value of such induced tetraploids in hybridization programs aiming to generate high pVAC triploid hybrids.

## Data Availability Statement

The datasets generated for this study are available on request to the corresponding author.

## Author Contributions

DA, ML, AB, and RS conceived and designed the study. DA conducted the study, performed the statistical analysis, and wrote the first draft of the manuscript. BM-D contributed to the carotenoid analysis. All authors contributed to the data interpretation, writing, and editing of the manuscript.

## Funding

This work was part of the Ph.D. research of DA, with financial support from HarvestPlus (www.HarvestPlus.org), a global alliance of agriculture and nutrition research institutions working to increase the micronutrient density of staple food crops through biofortification. The views expressed do not necessarily reflect those of HarvestPlus. This work was also supported by donors through their contributions to the CGIAR Fund (http://www.cgiar.org/who-we-are/cgiar-fund/fund-donors-2/) particularly to the CGIAR Research Program for Roots, Tubers, and Bananas (CRP-RTB).

## Conflict of Interest

The authors declare that the research was conducted in the absence of any commercial or financial relationships that could be construed as a potential conflict of interest.
